# Supplementary material for: Intrinsic Spine Dynamics Are Critical for Recurrent Network Learning in Models With and Without Autism Spectrum Disorder
Source: Front Comput Neurosci. 2019 Jun 13;13:38. doi: 10.3389/fncom.2019.00038 (PMC6585147; doi:10.3389/fncom.2019.00038)
Supplement: Supplementary file 1 [file Data_Sheet_1.docx]

**Supplementary Material**

Intrinsic spine dynamics are critical for recurrent network learning in models with and without autism spectrum disorder

James Humble, Kazuhiro Hiratsuka, Haruo Kasai, and Taro Toyoizumi

**
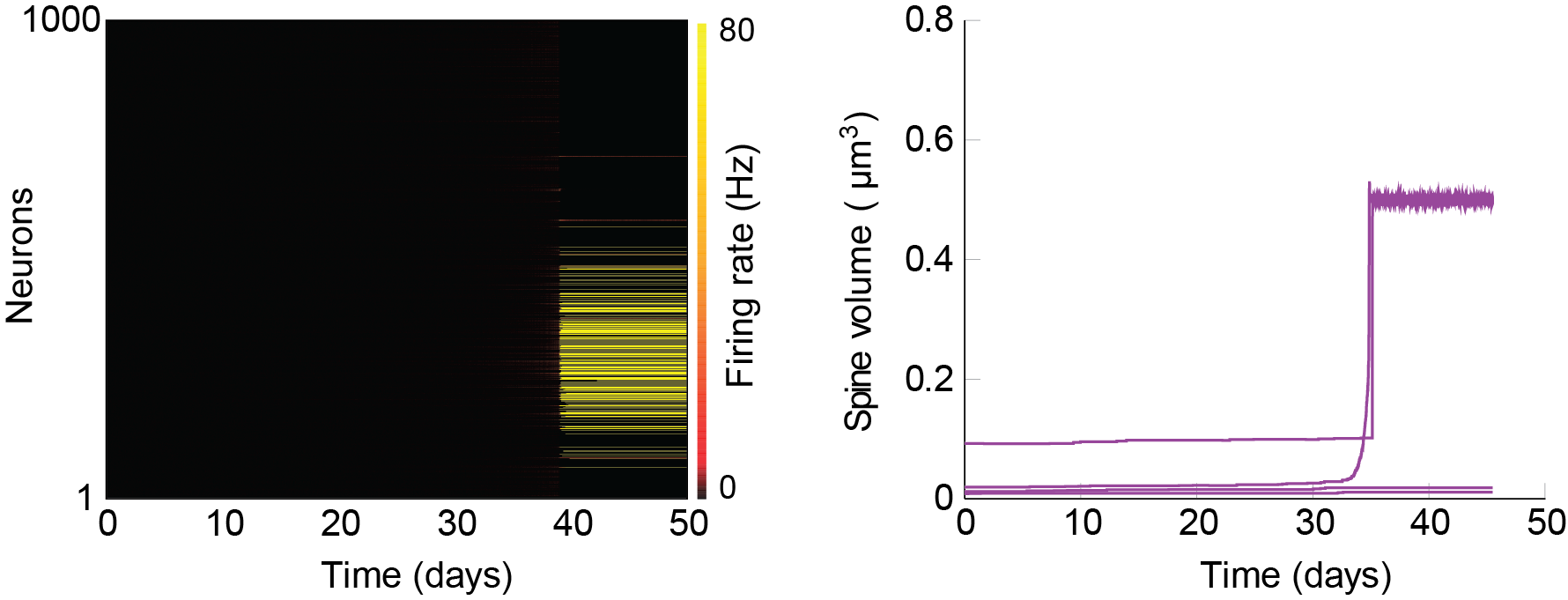
**

Figure S1: Network behavior without intrinsic spine dynamics and external stimulation. Note that the STDP learning rate was set 100 times greater than other simulations to expedite the process. (Left) Firing rate of excitatory neurons. The firing rates of many neurons exploded in the middle of the simulation. Some neurons have low firing rates because of the lateral inhibition from active neurons. (Right) Representative spine-volume traces. Spine volumes between active neurons converged to the prefixed LTP/LTD balancing point of the multiplicative STDP rule at around 0.5 µm^3^.


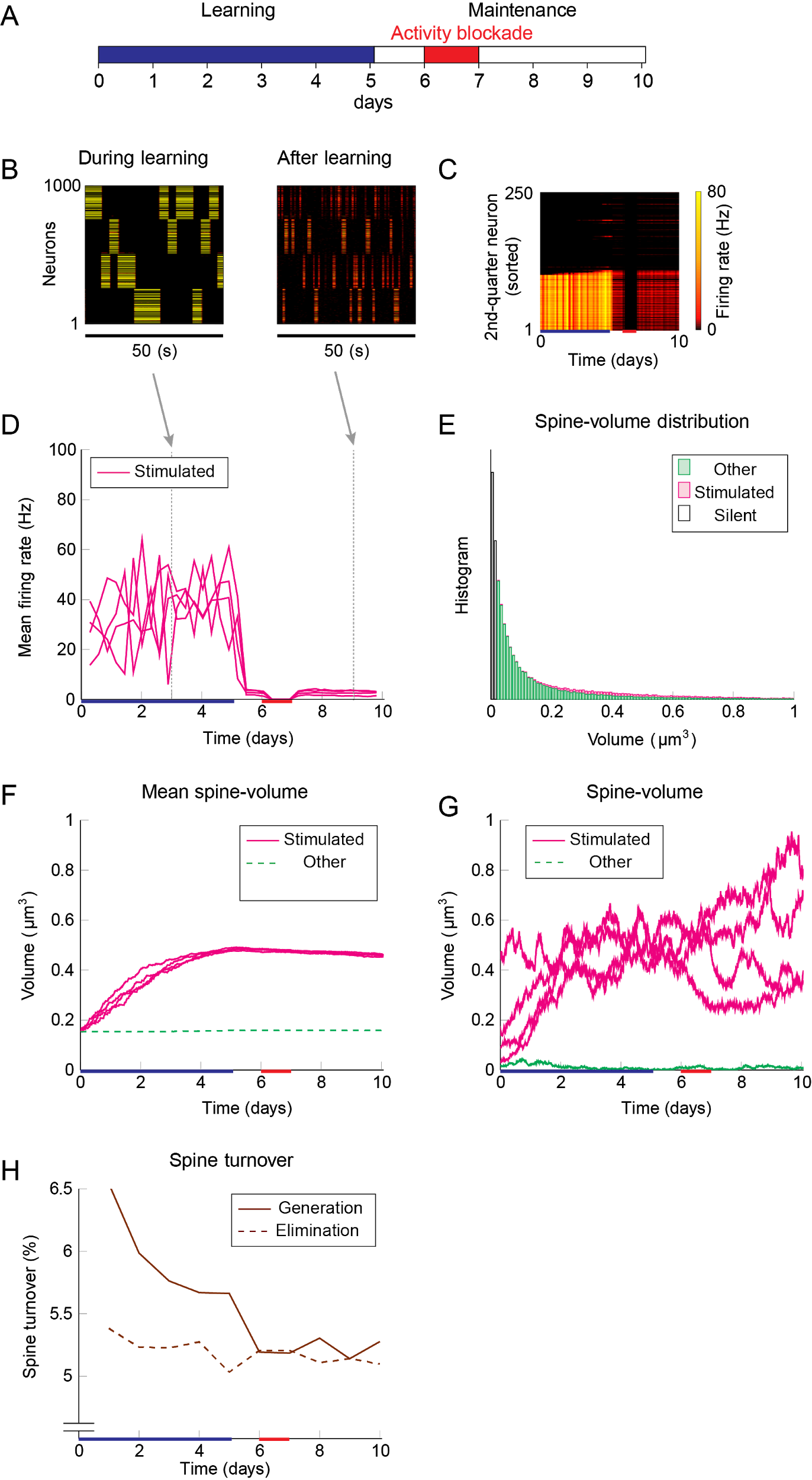


Figure S2: Network behavior in the presence of intrinsic spine dynamics, similar to Fig 3, but with 1-day blockade of neural activity during the maintenance period. The activity blockade does not change the results. (A-H) Conventions are as in Fig. 3.


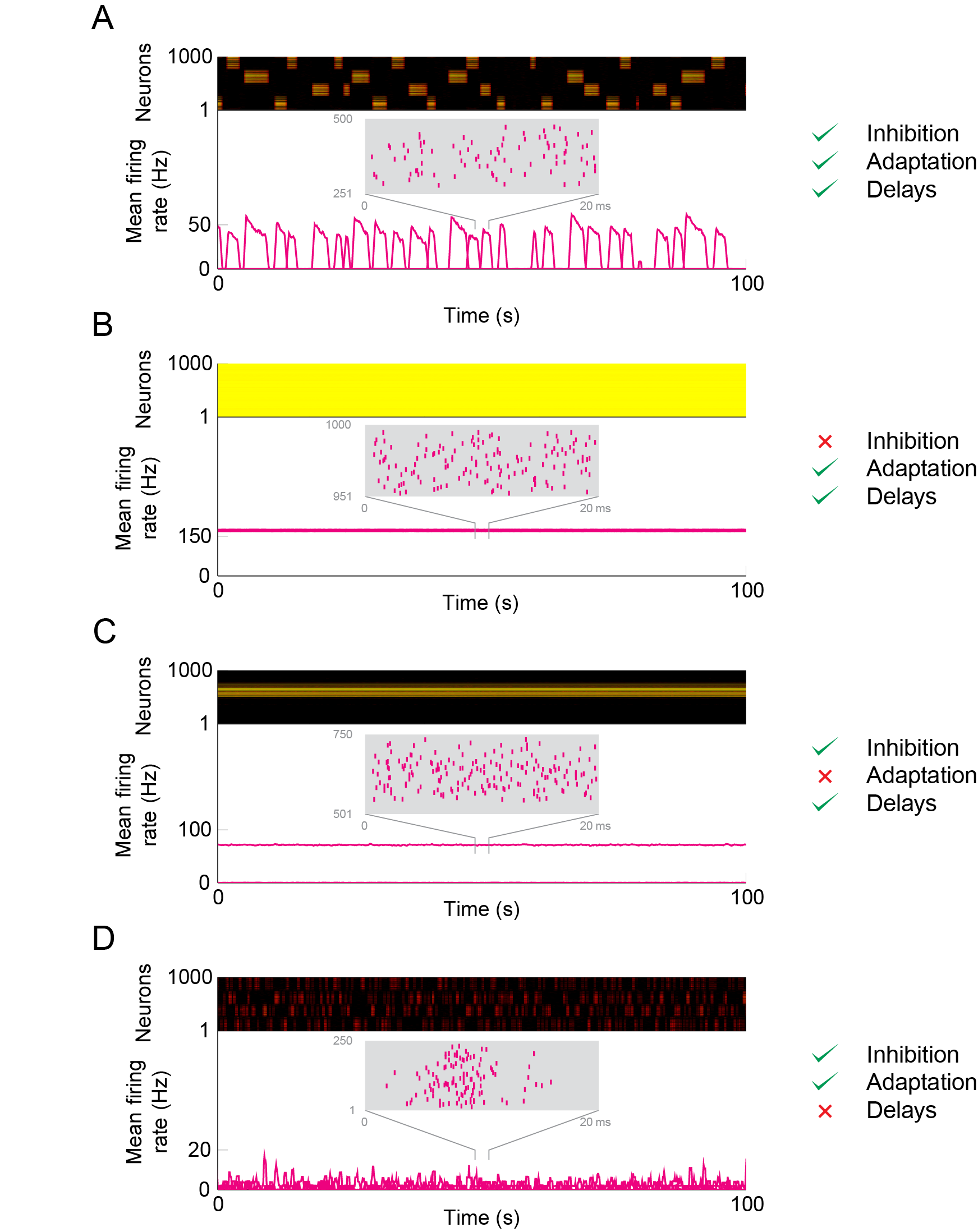


Figure S3: Description of different mechanisms in the model. (A) With inhibition, adaptation, and axonal delays all functioning, the network retains all cell assemblies where each assembly is rehearsed for several seconds during spontaneous activity. (B) When the inhibitory neurons are removed all excitatory neurons continuously fire a saturated rate >150 Hz. (C) When adaptation is removed from excitatory neurons, only one assembly dominates. (D) When axonal delays are removed, the four memories are somewhat maintained, albeit with very fast noisy switching and a much lower firing rate. The removal of a mechanism was done after learning and at the onset of the maintenance period, with all other mechanisms, including STDP and intrinsic spine dynamics, functionally preserved.

**
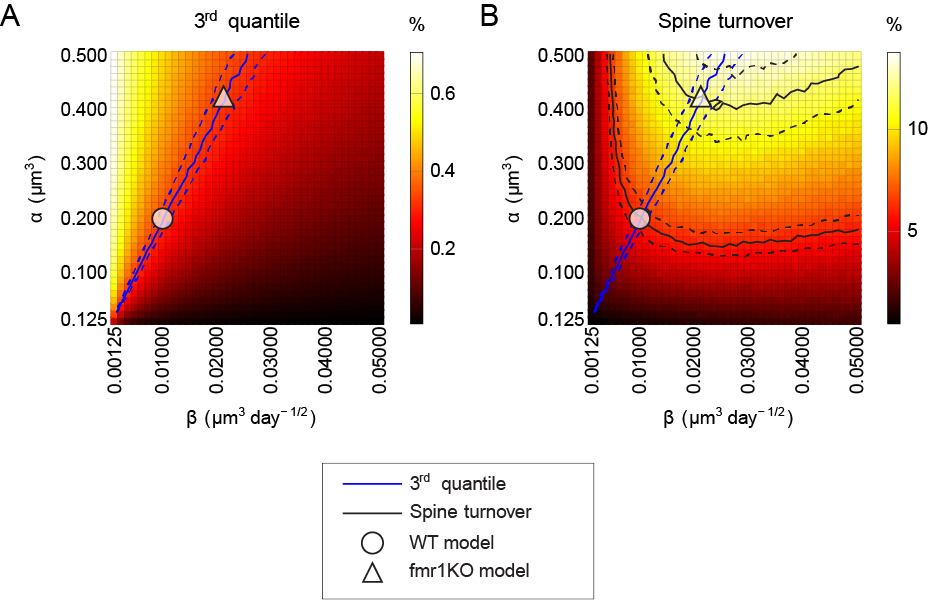
**

Figure S4: Systematic exploration of intrinsic spine dynamics’ parameters $\alpha$ and $\beta$. (A) The 3rd quantile of the equilibrium spine volume distribution is shown in color as a function of $\alpha$ and $\beta$. The entire distribution roughly scales with the ratio $\alpha/\beta$ as expected based on the theoretical consideration. The blue solid line (and dashed lines) indicates the experimentally observed 3rd quantile (and ±10% range). (B) Spine turnover is shown in color as a function of parameters $\alpha$ and $\beta$. Increases in either $\alpha$ or $\beta$ result in increases in spine turnover. The two solid black lines (and dashed lines) indicate experimentally observed spine turnover for WT and *fmr1*KO animals (and ±10% range). We therefore used the two parameter combinations of $\alpha$ and $\beta$at the cross points of the black and blue solid lines in our WT and *fmr1*KO models.
